# Energy Transfer to Molecular Adsorbates by Transient Hot Electron Spillover

**DOI:** 10.1021/acs.nanolett.3c00013

**Published:** 2023-04-03

**Authors:** Mirko Vanzan, Gabriel Gil, Davide Castaldo, Peter Nordlander, Stefano Corni

**Affiliations:** † Department of Chemical Sciences, University of Padova, Via Marzolo 1, 35131 Padova, Italy; ‡ Department of Physics, University of Milan, Via Celoria 16, 20133 Milan, Italy; § Instituto de Cibernetica, Matematica y Física, Calle E esq 15 Vedado, 10400 La Habana, Cuba; ∥ Department of Physics and Astronomy, Rice University, Houston, Texas 77005, United States; ¶ CNR Institute of Nanoscience, via Campi 213/A, 41125 Modena, Italy

**Keywords:** hot carriers, hot electrons, energy transfer, photocatalysis, electron dynamics, nanoplasmonics

## Abstract

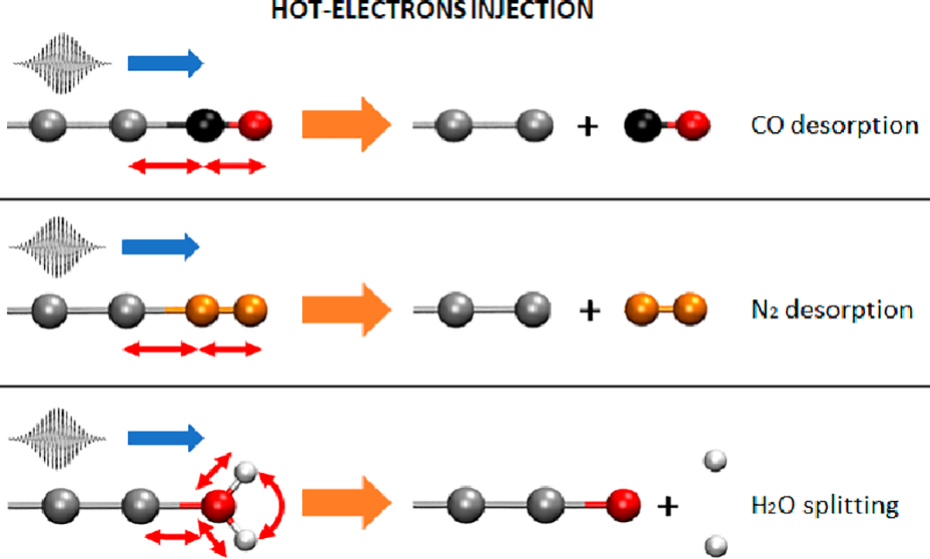

Hot electron (HE) photocatalysis is one of the most intriguing fields of nanoscience, with a clear potential for technological impact. Despite much effort, the mechanisms of HE photocatalysis are not fully understood. Here we investigate a mechanism based on transient electron spillover on a molecule and subsequent energy release into vibrational modes. We use state-of-the-art real-time Time Dependent Density Functional Theory (rt-TDDFT), simulating the dynamics of a HE moving within linear chains of Ag or Au atoms, on which CO, N_2_, or H_2_O are adsorbed. We estimate the energy a HE can release into adsorbate vibrational modes and show that certain modes are selectively activated. The energy transfer strongly depends on the adsorbate, the metal, and the HE energy. Considering a cumulative effect from multiple HEs, we estimate this mechanism can transfer tenths of an eV to molecular vibrations and could play an important role in HE photocatalysis.

Understanding and controlling light–matter interaction at the nanoscale is a key step for developing future technologies such as sensing, catalysis, renewable energy, and medicine.^1−3^

An important feature for the optical properties of metallic nanoparticles (NPs) is the Localized Surface Plasmon Resonance (LSPR), a collective oscillation of the conduction electrons. Due to its collective nature, the cross section for LSPR excitations can be very large compared to single electron excitations. LSPR can decay nonradiatively generating electron hole pairs, also referred to as hot carriers (HCs). By tuning the plasmon resonances to wavelengths where the nonradiative damping is large, plasmon excitation can be a very efficient source of HCs. This feature, which in the past was considered a detrimental phenomenon, has recently begun to be exploited in a wide range of applications.^4−7^

Nonradiative LSPR decay can be described as follows. The initial HC generation results in a nonthermal distribution of HCs: holes with energies ranging from *ϵ*_*F*_ – *ℏω*_*LSPR*_ to *ϵ*_*F*_ and electrons with energies ranging from *ϵ*_*F*_ to *ϵ*_*F*_ + *ℏω*_*LSPR*_, where *ϵ*_*F*_ is the Fermi level of the metal and *ℏω*_*LSPR*_ is the LSPR energy. This nonequilibrium distribution rapidly undergoes electron–electron interactions which result in HC multiplication and a decrease in the average energies of the electrons and holes. During this step, a small fraction of the HCs also relax radiatively through luminescence.^8^ In the latter part of this relaxation process (lasting up to ∼1 ps) the distribution of the HCs can be approximated as Fermi–Dirac distributions with high effective electron temperatures. On longer time scales, the electrons thermalizes with the phonons resulting in photothermal heating.^9−13^

There are many uncertainties regarding how HCs can enhance photocatalysis.^14−16^ Indeed, HCs may effectively interact with a molecule adsorbed on the NP.^17^ To date, several groups have been able to use HCs to induce different reactions, with higher selectivity and/or rates as compared with their thermal counterpart.^18−23^ It is worth mentioning that some of these HC catalyzed reactions, e.g., nitrogen fixation, water splitting, or carbon dioxide reduction, are extremely important from the technological point of view, especially considering the challenges set by global warming and climate change.^24^ HC photocatalysis can thus contribute to a green and sustainable future.^25−28^

Despite much progress on the characterization of these reactions from the experimental side, the way HCs interact with molecules and activate chemical processes is still a matter of debate.^29−31^ The two dominant mechanisms aiming to explain hot electron (HE) photocatalysis are the indirect and direct electron transfer. In the former, the HE is transferred to the lowest electronic energy level available (for simplicity, identified with the Lowest Unoccupied Molecular Orbital LUMO) of the target molecule. In the latter view, NP electrons are directly transferred into the LUMO state of the molecule via direct optical excitation.^5,9,17^ In both cases, HEs injection results in the weakening of intramolecular bonds and reduction of the energy barrier required for bond breaking and cannot happen if the LUMO level lies above *ϵ*_*F*_ + *ℏω*_*LSPR*_.

Although these mechanisms can explain a wide range of observations, they do not cover all possibilities for chemical transformations. For instance, HCs could in principle induce vibrational excitations in an adsorbed molecule and thereby influence its reactivity. This is particularly important since HC multiplication will result in a large number of lower energy HCs, each contributing to vibrational excitations. A few alternative ideas for explaining plasmon enhanced catalysis has been proposed such as near field enhancement effects^32,33^ or local increases of the temperature.^34−37^ None of these model the actual dynamics of HEs and their interactions with molecules.

Here we study HE induced energy transfer into molecular vibrational modes. In this mechanism, occurring after electron–hole pair thermalization via the carriers’ scattering, the HEs transiently spill out of the plasmonic nanostructure, interact with the adsorbed species, and destabilize its nuclear structures, releasing part of their energy to molecular vibrations. Therefore, the HEs act as external sources of energy that initiates and amplifies the vibrational oscillations. We will refer to this HE mediated energy transfer process as Vibrational Energy Transfer via Hot Electron (VET-HE). Such a mechanism has been proposed previously^31,38^ but to our knowledge not investigated in detail. Moreover, a recent experiment, has highlighted the primary role of molecular vibrations in the photoactivation of chemical processes, such as the Cu catalyzed H_2_–D_2_ exchange reaction.^39^

In contrast to the direct or indirect electron transfer approaches where molecular species are destabilized by discrete events, in VET-HE, reactions are promoted by continuous energy release from HCs to the molecule. The vibrational excitation results in faster bond breaking, which can be a rate-limiting step in chemical transformation and may be induced by HEs lying below the LUMO energy level. Such HE persist in the systems until their energy is dissipated via coupling with metal phonons, on longer time scales (∼1 ps) compared to the one investigated here (few fs).^2,9^ Metal phonons may also play a direct role in HE dynamics.^40^

To quantify the efficiency of the VET-HE effect, we performed *ab initio* calculations on simple atomistic models, consisting of a linear chain of metallic atoms and a molecular adsorbate tethered to one of its ends (see Figure 1a). The chain is a minimalistic model of a plasmonic system supporting HE propagation, whereas the terminal molecule is the target adsorbate to be excited by the HE. In these simulations, HEs were initially confined to the metal atom that is furthest away from the adsorbate and then allowed to move along the chain. We simulate the motion using real-time Time Dependent Density Functional Theory^41^ (rt-TDDFT) with clamped nuclei, as implemented in the code Octopus;^42−44^ see the Supporting Information for details. Adiabatic approximation is used here, a discussion on its reliability is given in the Supporting Information. Figure 1a shows a graphical representation of our model when silver is used as substrate.

**Figure 1:**
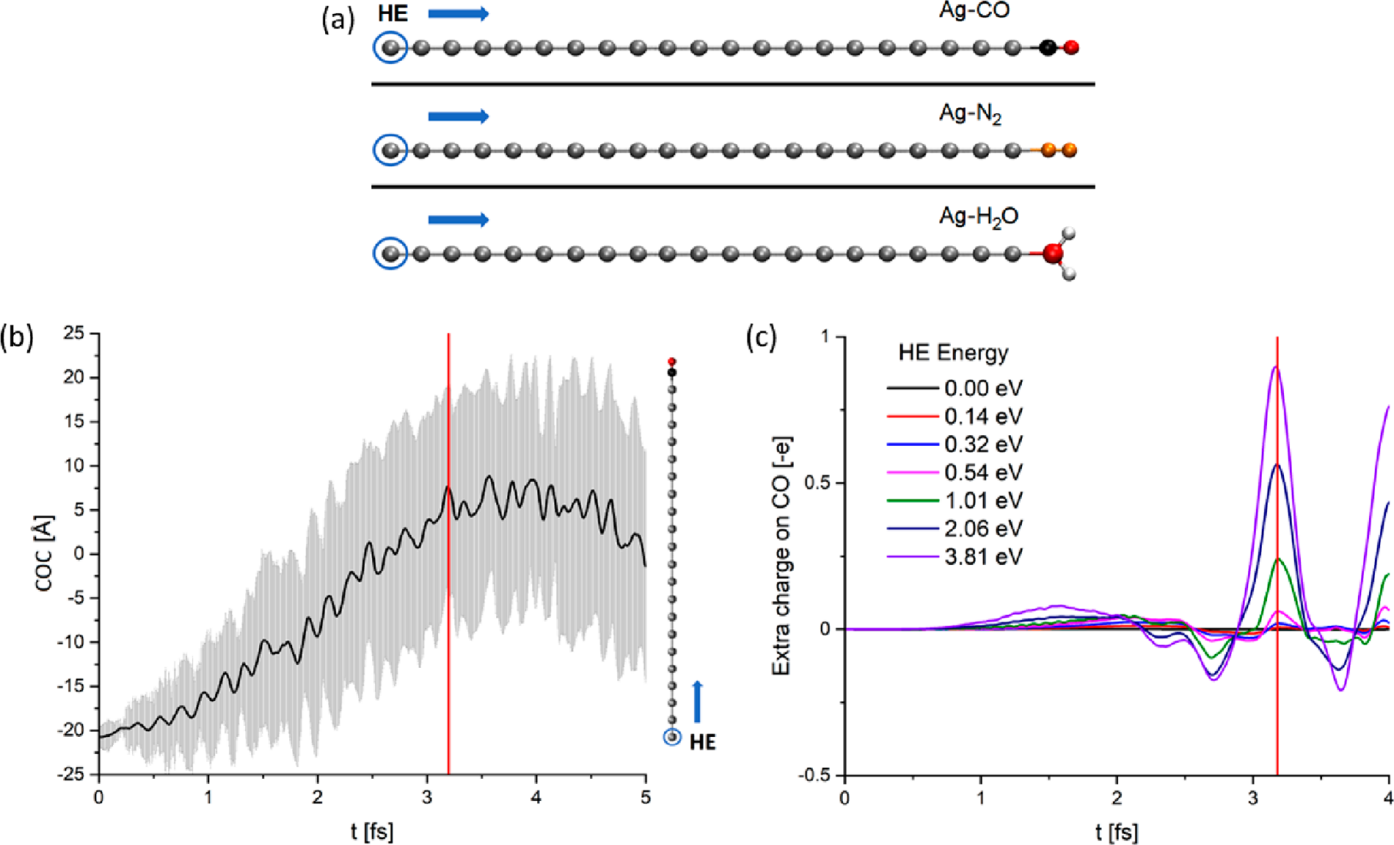
(a) Schematics of the models. The Ag chain stands for the metal substrate, and CO, N_2_, and H_2_O are the various adsorbates we explored. In each case, the blue circle marks the initial site for the HE, whereas the arrow shows the direction of HE motion. Gray, black, red, orange, and white balls represent Ag, C, O, N, and H atoms, respectively. (b) Time evolution of the COC for the Ag–CO system for a HE energy of 3.81 eV. The chain model on the right serves as a reference to understand the HE motion within the system. Gray lines represent the uncertainty connected to the HE position. (c) Amount of extra charges located on CO in the case of the Ag–CO chain, as a function of time and HE energy. In (b) and (c), the red line indicates the actual HE injection on the absorbed molecule (i.e., 3.2 fs).

By analyzing the amount of charge transfer and the forces acting on the molecule as a function of time, we were able to quantify how the HE interacts with the adsorbate, which vibrational modes are activated, and how these effects depend on the HE energy. We applied this method to different adsorbates (CO, N_2_, H_2_O) and substrates (Ag, Au). Due to the simplicity of the model, we are able to develop a clear picture of the overall process. Furthermore, our findings support recent evidence on hot carrier mediated reactions^45−47^ and suggest how HEs could improve the lifetime of photocatalysts in specific reactions.^48−50^ Compared to other approaches based on, e.g., the analysis of the optical responses,^51−56^ our method allows a simple and direct observation of the HE dynamics and gives insights on the energetics involved in the HE–molecule interaction.

We model the HE wave packet as the difference between time dependent and ground state electronic densities of the system. We now consider the evolution of the HE wave packet for the Ag–CO system (Figure 1a, top). Movie SM1 shows that the initially confined HE wave packet progressively spreads all over the system, gradually losing its initial coherence (identified here with the width of the wave packet). To quantitatively assess the motion of the HE, we monitor its average location and uncertainty using the Centroid Of Charges (COC) of the HE wave packet as defined in the Supporting Information. In Figure 1b, we report the COC evolution. The HE wave packet gradually shifts from a strongly confined configuration (at *t* = 0) to a state where the HE is homogeneously distributed along the chain. Indeed, as the COC moves toward the center of the chain, its positional uncertainty increases. Such dynamics is observed in all cases under study (see Figure S2), meaning that it is intimately connected to the physics behind the HE motions rather than to the chemical nature of the system.

From Figure 1b it appears that the COC position is proportional to the simulation time, suggesting a ballistic dynamics of the HE wave packet instead of a diffusive motion.^10,57−59^ This qualitative observation is confirmed by analyzing the root-mean-square deviation of COC data (see the Supporting Information), which linearly depends on *t* (ballistic dynamics) rather than on *t*^1/2^ (diffusive motion).^60^ From the size of the silver metal chain (i.e., 44.5 Å) and the time it takes the HE to reach the end of it (ca. 3.2 fs; see below), we estimate an average velocity for the HE of about 1.39 × 10^6^ m/s, which matches the Fermi velocity of conduction electrons in bulk silver.^61^ Such an agreement was obtained also for Au based systems (see the Supporting Information).

When the HE wave packet starts to interact with the adsorbate, the molecular charge density increases. Hence, we estimate the amount of transferred charge in a single injection by integrating the HE charge density at the adsorbate. As shown in Figure 1c, the charge transfer is strongly dependent on the energy of the HE wave packet. In particular, we can identify a major peak around 3.2 fs (red lines in Figure 1, panels b and c) corresponding to maximum charge transfer. The height of this peak depends on the original HE energy, but the peak itself is visible even for HE energies as small as 0.3 eV (see also Figure S4). Since our simulations do not account for nuclear motion, the transferred energy cannot be dissipated through electron–phonon interactions and therefore the physics following is less reliable. Beyond 3.2 fs, the HE motion loses its ballistic character (Figure 1b).

The presence of smaller peaks around 1.55 fs in Figure 1c, suggests that a charge transfer process may also have a long-range component, i.e., can change the density on the molecule even if the electron is still localized within the metal. In fact, around 1.55 fs the wave packet is still far from the adsorbate and moderately coherent. While we cannot rule out that this interaction is specific to the quasi one-dimensional nature of our model, it may be the first time long-range character of HE–adsorbate interactions (already observed in some devices^62,63^) are exposed in an atomistic simulation.

Figure 1c shows that, in addition to positive peaks, a charge-depletion area precedes the HE injection. This is particularly visible in the first frames of the movie SM1. The smooth decrease of electronic charge in the molecular region, occurring at 2.2–2.7 fs, represents a reverse charge transfer. In the future we plan to investigate the relation between the length of the chain and the frequency of charge oscillation on the molecule.

After the injection, the HE can interact with vibrational modes through the VET-HE mechanism. Thus, we now calculate the total amount of energy transferred to the adsorbate vibrational modes for each system as a function of HE energy (see the Supporting Information). The results are shown inFigure 2a,b. In particular, Figure 2a shows the results for two diatomic species (CO and N_2_) distinguishing between the activation of intramolecular stretching (dissociation mode, top panel) and the nanoparticle-adsorbate vibration (desorption mode, bottom panel).

**Figure 2:**
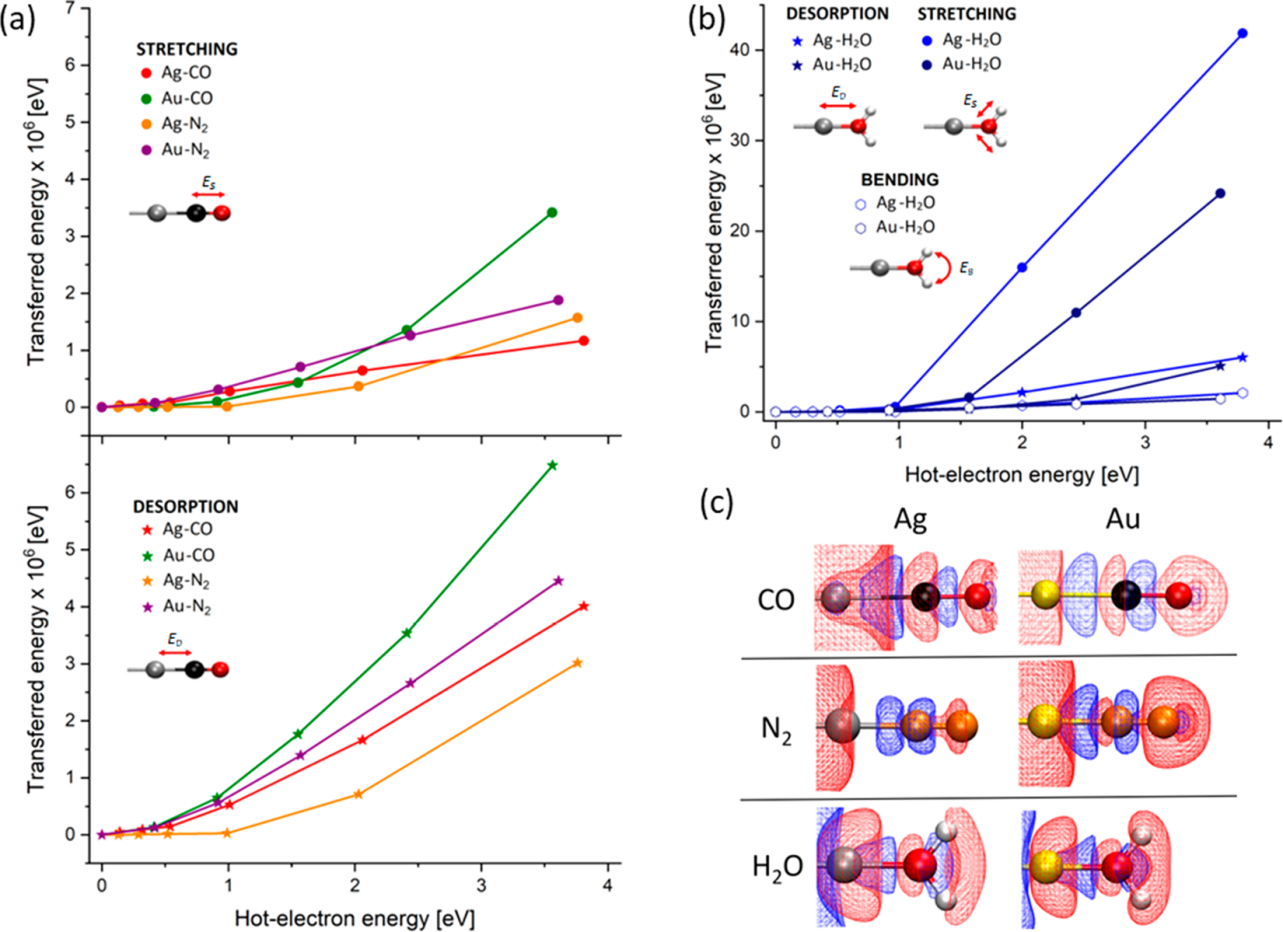
(a) Estimated transferred energy per HE injection, as a function of HE energy, in the case of M–CO and M–N_2_ systems (M = Ag, Au). The top panel refers to the intramolecular stretching modes while the bottom panel reports results on the activation of desorption modes. Inset pictures denote the two possible molecular vibrational motions. (b) Same as in (a), in the case of M–H_2_O systems (M = Ag, Au). Inset pictures denote the three possible molecular vibrational modes. In (b) and (a), solid lines are used as a guide to the eye. (c) Snapshot of the induced electronic density distribution at the time of maximal charge transfer during HE injection. Red and blue surfaces represent electron excess and depletion. Gray, gold, black, red, orange, and white balls represent Ag, Au, C, O, N, and H atoms, respectively. The HE energy is 3.56 eV for Au and 3.81 eV for Ag. Isovalues = ±10^–4^ e·Å^–3^.

The first aspect emerging from the plots is the correlation between the energy transferred and the HE energy. However, the energy transferred to the two modes is significantly different, and this may have profound consequences for the selectivity of HE catalysis. Most of the energy goes into the desorption mode, with a desorption-to-stretching transferred energy ratio spanning from 1.9 to 3.5, for HE energy around 3.5–4.0 eV. The effect, larger for Au than for Ag, has different consequences, depending on the reactions considered. Notably, even low-energy HEs (∼1 eV) can transfer energy to the target molecule.

For ammonia synthesis, N_2_ dissociation on the catalyst surface is the rate-limiting step so the use of Ag or Au nanoparticles as photocatalysts appears disadvantageous. Many efforts are currently being spent to find photocatalyst for N_2_ fixation that rely on other metals or composite materials.^64,65^ On the other hand, N_2_ desorption is the rate-limiting step in ammonia decomposition, which is a key reaction in processes that use NH_3_ as a medium for H_2_ storage and production.^45−47^ Our computational model suggests that HEs can enhance desorption, stimulating the molecular detachment.

Concerning CO, its desorption is a crucial step in methane steam reforming to prevent the water–gas shift reaction and coking,^48,66^ i.e., the formation of a layer of carbon atoms that poisons the catalyst.^49^ This is a major problem in the case of thermally activated methane steam reforming that can be reduced using photocatalysts exploiting hot carriers. Indeed, our calculations suggest that HEs may selectively activate the vibrations corresponding to CO desorption and this could reduce the coking rate. The exact energy to be transferred to achieve this goal depends on the details of the real material system considered, an estimate of the energy that can be transferred is given later on showing that an effect is possible.

Using a photocatalyst with high HC production rates and low affinity toward CO could therefore bring major improvements to the efficiency and sustainability of steam reforming processes. Conversely, for CO_2_ reduction, a photocatalyst with high CO affinity led to high reaction yields and selectivity toward reduction to CH_4_.^18,67−69^ Our evidence suggests that CO activation could be partially enhanced by excitation of C–O vibrational motion which, although weaker than the desorption motion, is still present.

A different picture emerges from the analysis of M–H_2_O systems (M = Ag, Au). As shown in Figure 2b, VET-HE predominantly stimulates the intramolecule symmetric O–H stretch mode. In this case, the stretching-to-desorption transferred energy ratio is about 7 for the Ag–H_2_O chain and 5 for Au–H_2_O in the 3.5–4.0 eV HE energy window. Larger numbers are obtained for the stretching-to-bending ratio, reaching 21 and 17 for Au and Ag cases, respectively. This indicates that, within our methodological framework, HE can selectively excite O–H symmetric stretching in water, and the magnitude of transferred energies are significantly larger than for the diatomic cases, the maximum values being 4.2 × 10^–5^ and 2.4 × 10^–5^ eV for Ag and Au, respectively. The predominant activation of such a mode is coherent with calculations on a charged water molecule (see the Supporting Information). The fact that HCs can effectively activate water splitting reactions is well established^70−73^ and a few computational studies were conducted to study the HC–water interaction.^53,56,74^ However, these studies focus on the charge transfer mechanism. Our approach represents a complementary mechanism where the O–H symmetric bond stretching activation could play a role in plasmon enhanced water splitting.

Interestingly, there is a correlation, although weak, between the strength of the adsorbate–metal bond and the amount of transferred energy by VET-HE (see the Supporting Information). The selective activation of specific vibrational motion can be understood by analyzing the molecular electronic density during the HE injection. In Figure 2c we show a snapshot of the HE-induced electronic density when the charge on the adsorbate is maximal (see the Supporting Information). In all cases there is a loss of electron density in the region between the metal and the molecule, suggesting a weakening of the metal–molecule bond, making the desorption more likely to happen.

There are some differences in the induced charge density between the systems. Regarding CO, we notice an electron density accumulation on top of the two atomic species, while the C–O bond region shows an electronic density depletion. This is valid for both Ag and Au based systems and indicates the C–O σ-bond orbital becomes depopulated in favor of the C–O σ* antibonding orbital, reflecting the activation of the C–O stretching. A similar effect occurs for H_2_O where for both Ag and Au, electron density is drained from the O–H internuclear region toward the atoms themselves, suggesting a weakening of the O–H bonds and thus an activation of the bond stretching. Lastly, in the case of N_2_, while the metal–nitrogen bond is weakened, the N–N bond is strengthened as suggested by the red wireframe surface surrounding the N–N bond, indicating increased electron population in the bond region. This suggests that the VET-HE mechanism is unlikely to result in N_2_ dissociation.

Finally, we provide an estimate of the energy that can be transferred to molecular vibrations by means of VET-HE in realistic systems. As shown in Figure 2a,b, the maximum amount of energy a single HE can transfer to a vibrational mode is in the order of 10^–6^–10^–5^ eV. However, in a real NP, multiple HC are generated from plasmon decay and from carrier multiplication;^8^ thus the energy transfer would be cumulative.

The total energy that can be transferred considering multiple injections can be expressed as
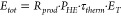
1where *R*_*prod*_ is the HC production rate which depends on the shape and nature of the NP,^75,76^*P*_*HE*_ is the probability of HE-molecule interaction, *τ*_*therm*_ is the thermalization time constant for a vibrational mode, and *E*_*T*_ is the energy transferred per injection event (10^–6^–10^–5^ eV from our estimate). If we apply eq 1 to the case of a CO molecule adsorbed on a cubic Ag NP (edge length 150 nm, illumination power 2.5 × 10^8^ W·cm^–2^), the *R*_*prod*_ is 10^17^ s^–1^.^77,78^ Assuming that the CO species is adsorbed on a nanoparticle hot spot, *P*_*HE*_ is around 0.5 (50% of HE are produced on the hot spots, see in particular Figure 7f of ref (79)). For the Ag–CO bond, a conservative estimation for *τ*_*therm*_ would be around 2 ps.^80^ Considering an *E*_*T*_ of 1.6 × 10^–6^ eV we get *E*_*tot*_ ≈ 0.16 eV, equivalent to an effective vibrational temperature *T*_eq,v_ ∼ 1900 K. This value is large enough to excite the vibrational levels of small molecules and thus to decrease the activation barrier for desorption (e.g., in the coking mentioned above). We note that the proposed VET-HE mechanism does not replace the others known mechanisms but can coexist and cooperate with them.

To summarize, here we present a novel mechanism for HE energy transfer into vibrational modes of a molecule chemisorbed on a metallic NP, VET-HE. No electronic excited states of the molecule need to be populated for VET-HE to occur. Such a mechanism has been validated from rt-TDDFT simulations. In particular, we show that the motion of the HE across the substrate can be viewed as ballistic with a gradual coherence degradation (spreading).

Most remarkably, we found that HE injection selectively activates specific vibrational modes involving both the nanoparticle-molecule and intramolecular vibrations, allowing energy transfer contributions from low-energy HEs.

The modes activation strongly depends on the molecule. In particular, we found that for CO and N_2_, desorption modes are activated more than the intramolecular vibrations, while in the case of H_2_O the activation of O–H symmetric stretching is largely favored over the other motions. These results are consistent with experimental observations^18,46,47,67,70−72^ and suggest that VET-HE mechanism may play a role in plasmon enhanced catalysis.

## Supporting Information Available

Movie of hot carriers dynamics in the case of Ag-CO (HE energy = 3.81 eV) (MP4)
Computational details and methods (graphical representation of the coupled harmonic oscillator models, table of spring constants); centroid of charges analysis; dynamics of centroid of charges motion; transiently transferred charge as a function of the HE energy; results obtained for Ag_7_–CO (amount of extra charges located on CO, estimated transferred energy per injection process); correlation between adsorption energy and HE-transferred energy (transferred energy per injection process vs adsorption energy); insight on the role of the computational approach (amount of extra charges located on CO, time evolution of the COC); analysis on the forces developing on charged water molecule (table of projection of the forces over the bending and symmetrical stretching motions); structural and geometrical parameters; description of C-EOM in the case of water molecule (PDF)
