# Supplementary material for: Energy Transfer to Molecular Adsorbates by Transient Hot Electron Spillover
Source: Nano Lett. 2023 Apr 3;23(7):2719–25. doi: 10.1021/acs.nanolett.3c00013 (PMC10103299; doi:10.1021/acs.nanolett.3c00013)
Supplement: Supplementary file 2 — nl3c00013_si_002.pdf [file nl3c00013_si_002.pdf]

Supporting Information

Energy transfer to molecular adsorbates by transient hot electron spillover

Mirko Vanzan,[†,‡] Gabriel Gil,[§] Davide Castaldo,[†] Peter Nordlander,[‖] and Stefano Corni[†,¶,*]

[†] Department of Chemical Sciences, University of Padova, Via Marzolo 1, 35131 Padova, Italy

[‡] Department of Physics, University of Milan, Via Celoria 16, 20133, Milan, Italy

[§] Instituto de Cibernetica, Matematica y Física, Calle E esq 15 Vedado 10400, La Habana, Cuba

[‖] Department of Physics and Astronomy, Rice University, Houston, Texas 77005, United States

[¶] CNR Institute of Nanoscience, via Campi 213/A, 41125, Modena, Italy

* email: stefano.corni@unipd.it

Computational details and methods

The NP-adsorbate hybrid systems were described at the atomistic level through one-dimensional models: 21-metal atoms chain made of Ag or Au, with CO, N_2 and H_2O molecules chemisorbed at one end (Figure 1a in the main text). The chain length was chosen so that the injected HE at time t=0 do not interfere with the molecule (they are separated by more than 40 Å). Moreover, an odd number of silver atoms allowed us to perform spin-unpolarized calculations, being the whole chain a closed shell system, saving a significant amount of computational resources. To ensure the size of the system itself does not influence the calculations, we tested the replicability of the results obtained within this framework by repeating the simulations for an Ag_7-CO system and verified that we got similar results as in the case of longer chain, (see next sections). To mimic the situation of a HE and arriving from elsewhere in the nanoparticle, all calculations accounts for an extra electron, so that all systems were negatively charged. Before performing the electron-dynamics calculations, all considered systems were optimized at the Density Functional Theory (DFT) level using the Local Density Approximation (LDA) exchange-correlation functional and applying a Broyden–Fletcher–Goldfarb–Shanno (BFGS) optimization procedure. During the optimization, the geometries were constrained along the directions orthogonal to the chain extension to preserve the one-dimensional nature of the systems. All the simulations were carried out using the package Octopus 10.4, which performs ab-initio calculations by computing the electronic density on a real-space mesh.[1,2] Both DFT and rt-TDDFT simulations were conducted on a space grid with a 0.12 Å spacing, contained within a simulation box built as the intersection of 6 Å radius spheres, centered on each atom. Obtained interatomic distances for the relaxed systems are collected in Table S1. The core electrons for the metal atoms were represented via Hartwigsen-Goedecker-Hutter (HGH) pseudopotentials[3], while for the non-metallic species we used standard pseudopotentials (psf files) as implemented in the code.

The starting configurations for TDDFT simulations, namely the state where HE were confined on the terminal atom of the chain, was obtained as the ground state electronic density of systems where the pseudopotential files of the terminal atoms were modified in order to deepen the potential well for the electrons. Such deepening of the HGH pseudopotentials were obtained by increasing the absolute values of C_1 and C_2 coefficients entering in the pseudopotential equation, defined according to the HGH equation presented in the original paper.[3] The energy of the HE was identified as the difference between the ground state energy of these "pseudo-modified" systems and the ground state energy of the optimized chains. A picture of the charge excess given by this modification is shown in the first frame of the movie SM1. The modified pseudopotential files were then replaced with the standard ones, using these out-of-equilibrium electronic density configurations as the starting point of a rt-TDDFT dynamics. These states were allowed to evolve by means of rt-TDDFT using an Approximated Enforced Time-Reversal Symmetry (AETRS) algorithm, selecting a timestep of 0.01 fs and a total simulation time of 5 fs, a time window long enough to observe the HE transport and injection processes, considering the length of our systems. As a first attempt we tried to perform the simulations explicitly accounting for the nuclei motion of by coupling rt-TDDFT with Ehrenfest dynamics, as implemented in the code.[1,2] However, these calculations were affected by numerical errors due to the presence of residual forces left from the geometry optimization of the systems, whose magnitude were comparable with the ones produced by the injection of a single HE.

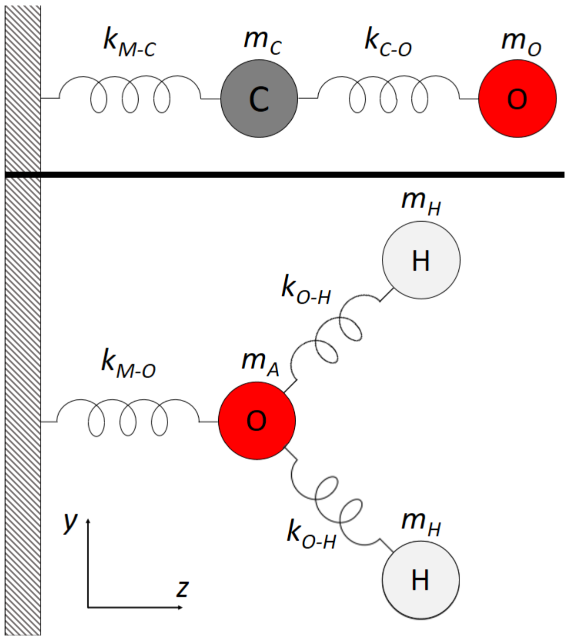

Figure S1. Graphical representation of the coupled harmonic oscillator models chosen to calculate the dynamics and energetics of the molecular systems along the simulations. Please notice that the uppermost model (CO) is analogous to the case of N_2 molecule, when masses and spring constants are properly chosen.

Therefore, the estimate of the HE-molecule transferred energy was conducted by modelling the molecular species as collections of coupled harmonic oscillators and recovering the energies through the integration Newton's second laws of motion i.e., the Classical Equations Of Motions (C-EOM). In this approach the molecular systems are represented by a set of classical particles and harmonic bonds whose masses and force constants were the ones of the atomic species (C, O, H and N) and the relative bonds (C≡O, O-H, N≡N), while the metallic atoms were considered static due to their relatively larger mass. The metal-atomic species M-X bonds (where M=Au, Ag and X=C, O, N) were also modelled considering the force constants available in the literature. See Figure S1 for a representation of these models. In the case of CO species, the C-EOM for the atoms can be written as:

$$\begin{cases} m_C \ddot{z}_C(t) + k_{M-C} z_C(t) + k_{C-O}[z_C(t) - z_O(t)] = F_C^z(t) \\ m_O \ddot{z}_O(t) - k_{C-O}[z_C(t) - z_O(t)] = F_O^z(t), \end{cases} \tag{S1}$$

where m_C, m_O, are the masses of atoms C and O, k_{M-C}, k_{C-O} are the spring constants of the M-C (M=Au, Ag) bond and intramolecular C≡O bond, $z_C(t)$, $\ddot{z}_C(t)$, $z_O(t)$, $\ddot{z}_O(t)$ are the variation of the positions and accelerations of atoms C and O given with respect to their initial values, and $F_C^z(t)$, $F_O^z(t)$ are the forces acting along the z axis on C and O at time t and due to the time dependent electron density. We considered only the z-projection of the forces because, since both the metal chains and the CO molecule extend along the z axis, the forces coming from the electron density have finite components along the z direction only. By a proper substitution of the parameters in equations (S1), we could represent the C-EOM also for the N_2 case, since it has the same geometrical configuration of CO (see Figure S1). A complete description of C-EOM in the case of water molecule is available in the following sections. The coupled harmonic oscillator C-EOM have been solved numerically by means of a first order Euler integration method allowing the computation of the displacements for all the atoms. Atomic masses were collected from the latest release of the official IUPAC periodic table of elements[4] while the values chosen for the spring constants are collected in Table S1. Here, M is the metal while X and Y are the atoms constituting the various molecules. Notice that the force constants in the case of M-N_2 calculations are the same for both M=Au, Ag. This because N_2 does not adsorb on Au and therefore an estimation of k_{Au-N} in the case of N_2 species would be uncertain.[5,6] Moreover, we encountered severe convergence problems in the optimization of the Au-N_2 geometry, since N_2 naturally tends to move away from the terminal gold atom. This prevents us from obtaining a proper geometry in this case and thus we chose to model the system imposing the Au-N and N-N distance as in the Ag-N_2 case. For these reasons, we decided to chose $k_{Au-N} = k_{Ag-N}$ so that we could at least qualitatively compare the performances of the two metals toward the dissociation of this molecule.

Table S1. Spring constants for the various M-X and X-Y intramolecular bonds. M=Ag, Au; X=C, N, O and Y=O, N, H. All values are given in N/m. [a] = taken from ref.[7] ; [b] = taken from ref.[8] except for the Au-N and N-N case, which values come from ref.[7]

M	X	k_{M-X}	X-Y	k_{X-Y}
Ag[a]	C	31	C-O	1822
	N	20	N-N	2407
	O	84	O-H	813
Au[b]	C	46	C-O	1589
	N	20	N-N	2407
	O	96	O-H	796

The energy transferred by HEs to the vibrational modes was calculated, as the sum of the kinetic and potential energy associated to the particular vibrational motion. Considering the CO case, these quantities were defined as:

$$E_D = \frac{1}{2} m_{CO} [\dot{z}_{CO}(t)]^2 + \frac{1}{2}\ k_{M-C} [z_C(t)]^2 \quad \text{(S2.1)}$$

$$E_S = \frac{1}{2} \mu_{CO} [\dot{r}_{CO}(t)]^2 + \frac{1}{2}\ k_{C-O} [r_{CO}(t)]^2 \quad \text{(S2.2)}$$

Where E_D, E_S are the energies transferred to the desorption vibrational motions (the one involving the M-C bond) and the intramolecular C-O stretching motion respectively, m_{CO} is the mass of the CO molecule, $\dot{z}_{CO}(t)$ is the first derivative of the C-O center of mass displacement with respect to time, μ_{CO} is the reduced mass of the CO molecule, $r_{CO}(t)$ is the calculated intramolecular bond length defined as $r_{CO}(t) = z_C(t) - z_O(t)$ and $\dot{r}_{CO}(t)$ is its derivative with respect to time. Also here, by properly substituting the parameters in equations (S3.1) and (S3.2), we could compute the transferred energies for the N_2 case. For H_2O desorption we have an analogous equation for the energy transferred to the desorption motion:

$$E_D = \frac{1}{2} m_{H_2O} [\dot{z_{H_2O}}(t)]^2 + \frac{1}{2}\ k_{M-O} [z_O(t)]^2 \quad \text{(S4.1)}$$

On the contrary, for internal vibrations, the equations are slightly more complicated since the O and H relative motions can be associated to both O-H symmetric stretching and H_2O scissoring (bending) motions. Asymmetrical stretching is not accounted since transfer of energy to that motion is forbidden by the symmetry of the system. To distinguish between the two internal motions, we computed the displacement vector associated to the H_2O molecule at the DFT level, as implemented

in the code Gaussian16.[9] We performed this calculation at the LDA/6-31+G* level and recovered the normalized vibrational displacement vectors for the symmetric stretching and bending motions that from now on will be called $\overline{\boldsymbol{d_S^\iota}}$ and $\overline{\boldsymbol{d_B^\iota}}$ where *i* indicates the O and H species**.** We then computed the projections of the relative atomic forces over the vibrational displacements vectors as follows:

$$F_S^i(t) = [\overline{\boldsymbol{F^\iota(t)}} \cdot \overline{\boldsymbol{d_S^\iota}}] \tag{S4.2}$$

$$F_B^i(t) = [\overline{\boldsymbol{F(t)}} \cdot \overline{\boldsymbol{d_B}}] \tag{S4.3}$$

Where $F_S^i(t)$ and $F_B^i(t)$ are the projection of the forces acting on the atom *i* over the normalized vibrational displacements related to symmetric stretching and bending respectively. We then express the obtained forces on the basis of the cartesian coordinates and used them to solve the C-EOM. This allowed us to obtain the atomic displacements relative to the two possible vibrational motions in cartesian coordinates. We then calculated the transferred energy as:

$$E_S = \frac{1}{2}\mu_{OH}\left[\dot{r}_{OH}^{S}(t)\right]^2 + \frac{1}{2}\, k_{O-H}[r_{OH}^S(t)]^2 \tag{S4.4}$$

$$E_B = \frac{1}{2}\mu_{OH}\left[\dot{r}_{OH}^{B}(t)\right]^2 + \frac{1}{2}\, k_{O-H}[r_{OH}^B(t)]^2 \tag{S4.4}$$

Where $r_{OH}^S(t)$ and $r_{OH}^B(t)$ are the relative variation of the normal coordinates of the vibrational mode (namely the O-H bond length and the H-H bond length for $r_{OH}^S(t)$ and $r_{OH}^B(t)$ respectively) calculated from the atomic displacements coming from the resolution of the C-EOM system. Please notice that we did not explicitly include any spring modelling the H-H interactions (see Figure S1). This assumption is reasonable considering that the largest majority of the HE energy is transferred to the stretching motion, as discussed in the main text. Finally, we remark that in all cases the predominant energy transfer is into kinetic energy term, being generally 10^2-10^3 times larger than the energy transfer into potential energy. This is because while a single injection process does not change much the atomic positions within its short interaction time (changes are on the order of 10^{-5}-10^{-6} Å) the momentum transfer is significant and the velocities can reach around 10^{-4} Å/fs in the case of the O-H bond. By integrating the electronic densities within the volume of the box corresponding to the molecule with respect to the simulation time and comparing these results with the transferred energies, we were able to estimate the instant at which the HE is transiently injected in the molecule and thus the amount of transferred energy connected to its presence. The estimation of the amount of charge located on the molecule as a function of time through integration of the electronic density, were performed as follows:

$$Charge(t) = \int_{V_m} \rho(x,y,z,t) - \rho_{GS}(x,y,z)\, dV_m \tag{S5}$$

where V_m is the volume of the region containing the molecule defined as the volume ranging from the plane orthogonal to the z axis and bisecting the M-X bond to the terminal part of the simulation box, $\rho(x, y, z, t)$ is the electronic density of the system at time t and $\rho_{GS}(x, y, z)$ is the electronic density of the ground state of the system. To monitor the position of the HE along the chain extension in time, we calculated the Centroid Of Charge (COC) as a function of time as follows:

$$COC(t) = \frac{\int_V z\,[\rho(x,y,z,t) - \rho_{GS}(x,y,z)]dV}{\int_V [\rho(x,y,z,t) - \rho_{GS}(x,y,z)]dV} \quad \text{(S6)}$$

where V indicates the volume of the whole simulation box and z is the coordinate along the chain axis. Notice that the difference between the two electronic densities is maximum when t=0 since here the HE confinement is maximum. The spread of the HE wave-packet is then calculated as the root mean square deviation around the COC(t), expressed as follows:

$$RMSD\,(t) = \sqrt{\frac{\int_V [z - COC(t)]^2\,[\rho(x,y,z,t) - \rho_{GS}(x,y,z)]dV}{\int_V [\rho(x,y,z,t) - \rho_{GS}(x,y,z)]dV}} \quad \text{(S7)}$$

All systems images, including the ones picturing the electronic densities, were produced using the VMD visualization package.[10]

Centroid Of Charges (COC) analysis

We reported in Figure S2 the evolutions of COC with respect to the time for the tested systems. In all cases, the results refer to the simulations where the HE energy is maxima (3.81 eV in the case of Ag-N_2 and Ag-H_2O, 2.0.3 eV for Ag_7-CO and 3.56 eV in the case of Au based systems). Red lines indicate the moment in which the HE is injected in the molecule. This time is 3.2 fs in the case of Ag substrate and 2.75 fs in the case of Au-supported systems. This time difference is not related to the metal itself (the fermi velocity of Ag and Au are almost identical[11]) but to the different metal-metal bond length within the chain that makes gold-based chains shorter than the silver systems (see Table S3 and the “Structural and geometrical parameters” section for more details). The moments in which the injection processes occur are fully compatible with the estimations that can be made on the basis of the systems Fermi velocities. Notice that in the case of Ag_7-CO (top left panel of Figure S2), the injection takes place in a sensibly shorter time, since the chain is shorter compared to all other systems (it is made by 7 metal atoms while all others are 21-atoms long).

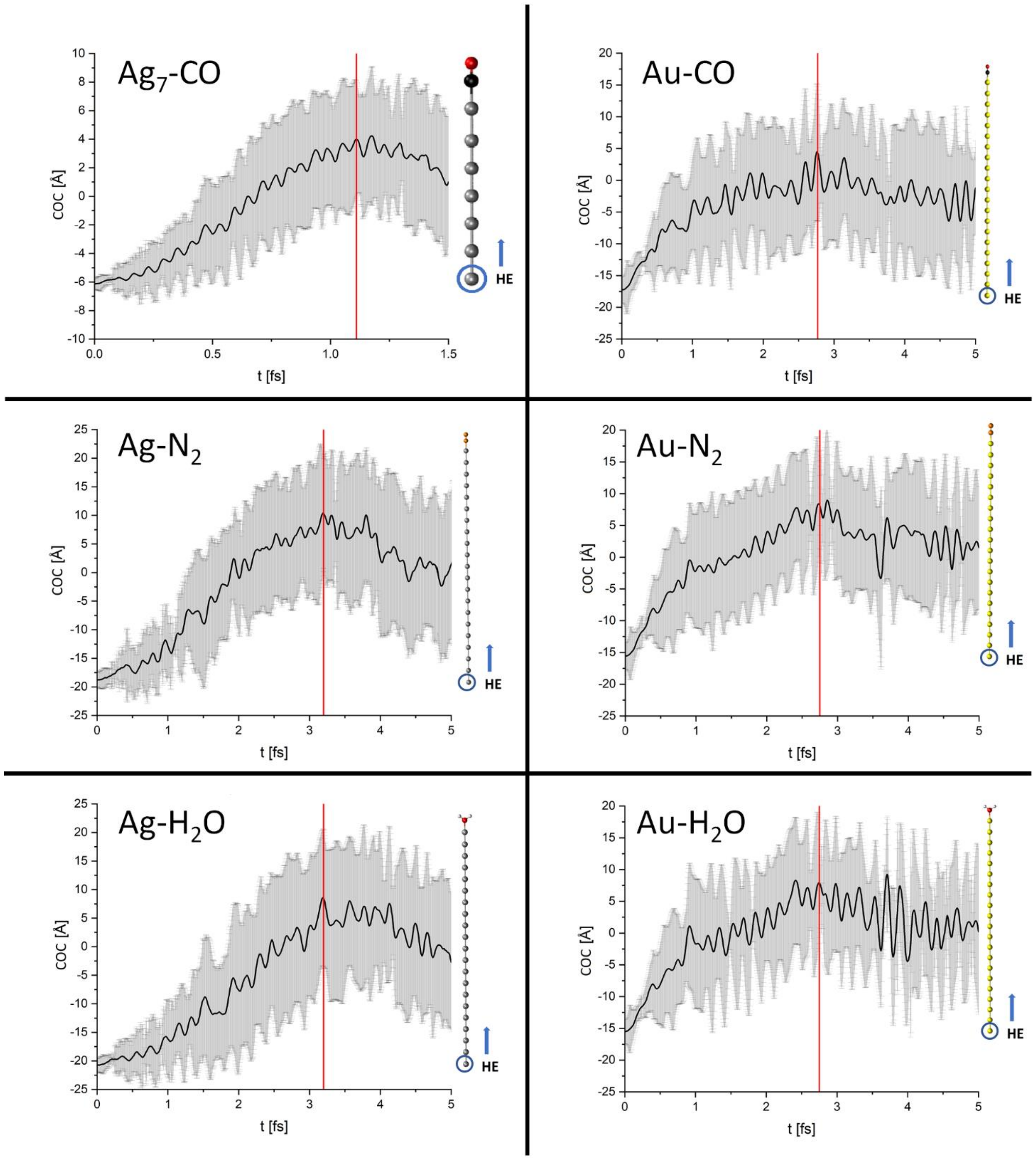

Figure S2. Centroid Of Charges (COC) analysis of all tested systems, when the energy of the adopted HE is maximum. Red lines indicate the moment in which the charge on the molecular species is maximum, namely the moment when the injection process occurs. Graphical representation of the model systems served as a guide to understand the HE wave-packet displacement along the system extension.

Dynamics of Centroid of Charges motion

In order to recover if the HE dynamics resemble more a ballistic or a diffusive motion, we performed linear regressions of the COC root mean square deviation with respect to its initial position, as a function of time and a function of the square root of time. Such quantity σ(t) is therefore defined as follows:

$$\sigma(t) = \sqrt{\frac{\int_V [z-z_0]^2\ [\rho(x,y,z,t) - \rho_{GS}(x,y,z)]dV}{\int_V [\rho(x,y,z,t) - \rho_{GS}(x,y,z)]dV}} \qquad \text{(S1)}$$

Where z_0 is the position of COC calculated at t=0. The plots are collected in Figure S3, panel A and B respectively. These regressions can unravel the type of motion observed in the simulations since ballistic and diffusive motions are characterized by a different dependence of the displacements on time. In particular for ballistic motion the relation between displacement and time is linear, while is a square root in the case of diffusive motion. As noticeable by the R-squares of the two fits, the data demonstrate that HE dynamic has to be interpret more as a ballistic motion than a diffusive one.

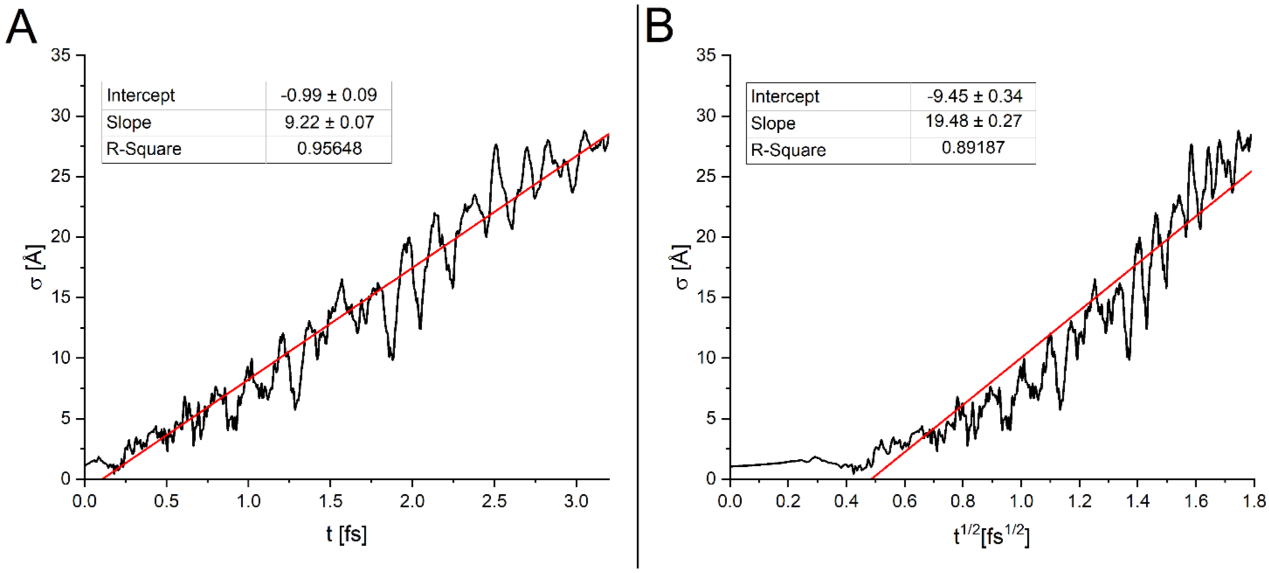

Figure S3. Panel A: Linear fit of σ(t) as a function of time. Panel B: Linear fit of σ(t) as a function of the square root of time. The data come from the same simulation, where the HE energy is 3.81 eV.

Transiently transferred charge as a function of the HE energy

As mentioned in the main text, the amount of charges that transiently localizes on the adsorbate depends on the original HE energy. This is clearly visible Figure S4 where we reported the maximum amount of charge that is transferred to the CO molecule in the Ag-CO simulations, as a function of the HE original energy. The charge values are taken at 3.2 fs, which is the moment in which the HE injection occur according to the graph in Figure 2b. Notice that there is a direct proportionality among the initial HE energy and the charge transferred to the adsorbate.

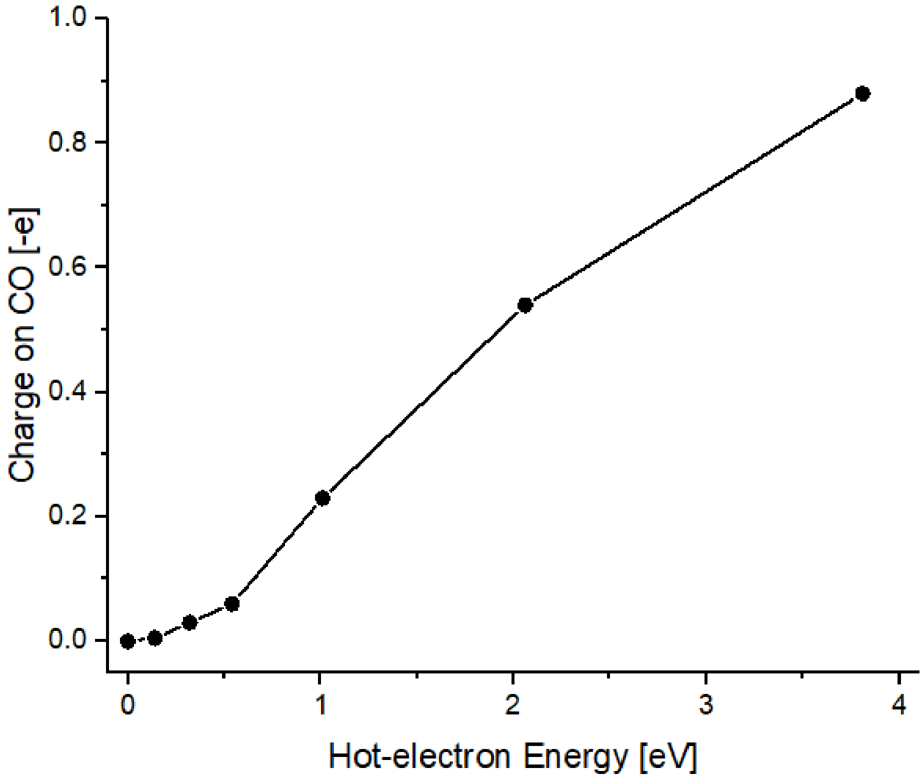

Figure S4. Amount of extra charges located on CO in the case of Ag-CO model short-chain system, as function of the HE energy. The values are taken at t=3.2 fs, namely the moment when the injection actually take place. Solid line have to be used as a guide for the eye.

Results obtained for Ag_7-CO

As mentioned in the main text, we performed the calculations on a shorter system, to assure the dimension of the chain itself does not affect the simulations results. We therefore apply our methodology to a chain-molecule system composed on a CO molecule adsorbed on a 7-atoms silver chain. The COC motion when the HE energy is 2.03 eV is pictured in Figure S2. Such HE energy was the larger we could simulate for such a small system (calculations accounting for higher HE energy did not converge). As visible from the COC trend, this follows the expected dynamics, being the HE confined on the last atom of the silver chain (around -6 Å) and gradually moving towards the other side of the system, while increasing its delocalization. The charge analysis in Figure S5 clearly measure a growing of the charge density on the molecule as the simulation proceeds, showing maxima at 1.1 fs. Considering that the chains is c.a. 13 Å long, this result is compatible with the fermi velocity of silver, as well as for the other systems.

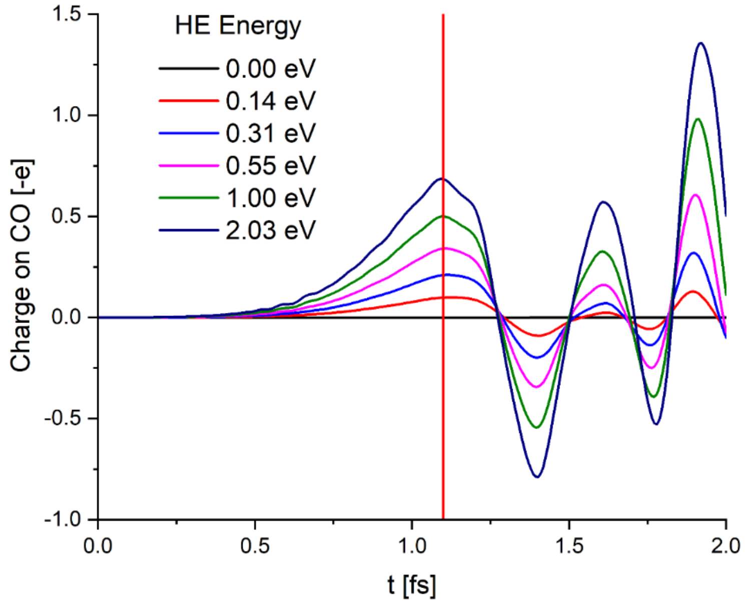

Figure S5. Amount of extra charges located on CO in the case of Ag_7-CO model short-chain system, as function of time and HE energy. Red line indicates the moment identified as the actual HE injection on the absorbed molecule (1.1 fs)

The HE-molecule transferred energy analysis reveal a dependence on the HE energy fully compatible with what observed in the case of longer 21-metal atoms Ag-CO system, as shown in Figure S6. Minor differences can be related to the smaller delocalization the HE wave-packet can have in this smaller system, which makes the

energy transfer more effective. However, such deviations are almost negligible, suggesting that our simulations are not sensibly influenced by the choice of the chain length, at least in the 7-21 metal atoms range.

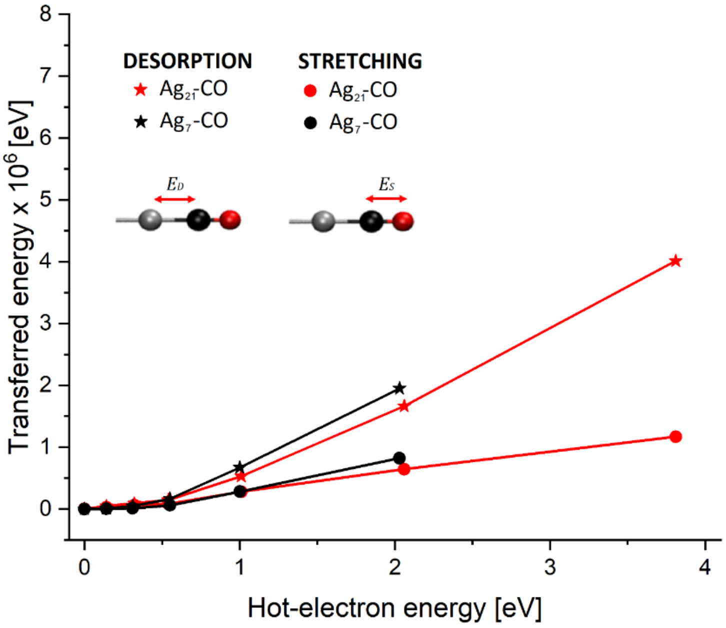

Figure S6. Estimated transferred energy per injection process, as a function of HE energy, in the case of Ag_{21}-CO (main model) and Ag_{21}-CO (short-chain model). Inset pics denote the two possible molecular vibrational motions. Solid lines have to be used as a guide for the eye.

Correlation between adsorption energy and HE-transferred energy

In the main text we demonstrated that the energy transferred to the different vibrational modes depends on the nature of the molecular species adsorbed on the metal chain. Here we further explore this aspect by investigating how the energy that can be transferred through the VET-HE mechanism depends on the strength of the metal-molecule bond. To do that we collected all data coming from both Ag and Au-based systems, in the case where the HE has its maximum energy (3.56 eV for Au and 3.81 eV for Ag), and plotted them as a function of the molecular adsorption energy. The latter quantity was calculated using the following equation:

$$E_{ads} = E_{chain} + E_{mol} - E_{sys} \tag{S8}$$

Where E_{chain} is the total energy of the metal chain, E_{mol} is the total energy of the molecule in vacuum and E_{sys} is the total energy of the systems where the molecule is adsorbed on the metal chain. All calculated E_{ads} values, are collected in Table S2.

Table S2. Calculated adsorption energy ($\boldsymbol{E_{ads}}$)and amount of HE-transferred energy (HE-TE) to the desorption (D) and inner stretching (S) vibrational motions, for the various M-m systems. M=Ag, Au; m=N_2, CO, H_2O.

M	m	E_{ads} [eV]	D HE-TE x 10^6 [eV]	S HE-TE x 10^6 [eV]
Ag	N_2	0.86	2.9	1.6
	CO	1.44	4.1	1.1
	H_2O	2.45	6.3	42
Au	N_2	0.55	4.4	1.7
	CO	1.12	6.5	3.5
	H_2O	2.12	5.1	24

From the plot in Figure S7, it can be seen how the energy transferred appears to be positively correlated to the strength of the metal-molecule bond, regardless of the considered vibrational motion. However, exceptions to this trend are present. In particular we found that in the Au-H_2O case the energy transferred to the desorption motion is slightly less than in the Au-CO case, where the metal-molecule interaction is weaker. The same feature is observed considering the activation of the inner stretching motion in the Ag-CO and Ag-N_2 systems.

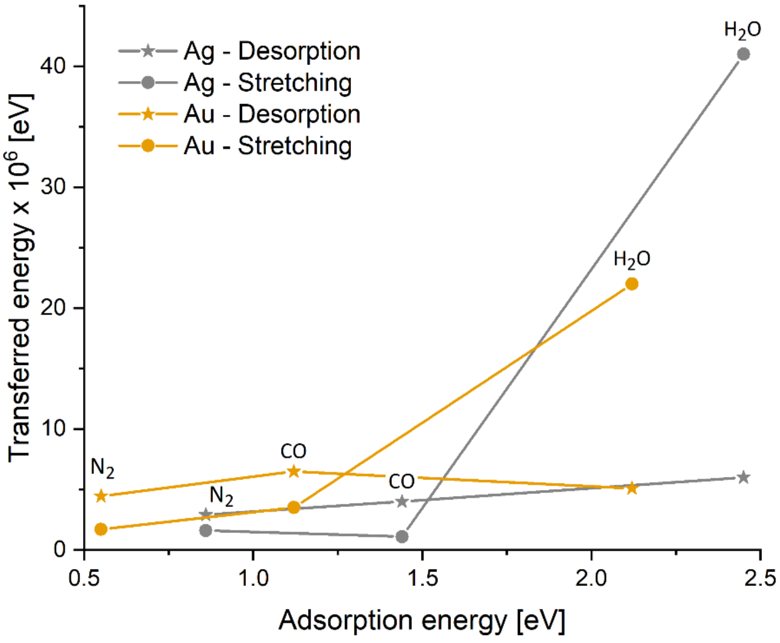

Figure S7. Transferred energy per injection process, as a function of the metal-molecule adsorption energy, in the case where the HE has its maximum energy (3.56 eV for Au and 3.81 eV for Ag). Inset labels, together with the symbol colors denote a particular metal-molecule system. Solid lines have to be used as a guide for the eye.

Therefore, although a correlation seems to exist, we do not judge our results solid enough to draw realistic conclusions on this point. This last statement is especially true if we consider that the adsorption energies we calculated refer to model systems and thus do not accurately represents any realistic photocatalytic device.
As an example, we invite the reader to consider that while it is known that N_2 does not adsorb on Au extended surfaces, we find a non-zero adsorption energy in our Au-N_2 system.

Insight on the role of the computational approach

In order to validate the quality of the chosen computational protocol, we performed another calculation on the Ag-CO system, using the Perdew–Burke–Ernzerhof (PBE) approximation of the XC-functional.[12]
In Figure S8 we reported the analysis of the extra charge on CO as a function of time, while the evolution of the COC is shown in Figure S9. In this PBE-based simulation the HE energy is 3.64 eV, thus these results have to be be compared with the simulation discussed in the main text where the HE energy is 3.81 eV, which results are visible in Figure S8 and S9 and labelled as LDA. Looking at Figure S8 it is clear how the time evolution of the extra charge present on the molecule is similar to the one obtained with the LDA XC-functional, i.e., the one discussed in the main text (see Figure 1c). In the PBE simulation the first notable peak occurs at 3.15 fs (blue line in Figure S8), which is comparable to the 3.2 fs obtained in the LDA case. The main slight difference resides in the maximum value of this peak which in the simulation performed with PBE reaches -1.13 e, while in the LDA case this value is around -0.80 e. Even the dynamics of the HE itself seems not to be remarkably affected by the choice of the XC-functional, as visible from Figure S9. The COC evolution is indeed very similar to the one of the LDA case (black line in Figure S9 and in Figure 1b). Here the only notable difference resides in the wideness of the wave-packet delocalization. Indeed it seems that using PBE the HE wave-packet remains more coherent along the dynamics as the associated error bars are narrower in the considered time interval. Finally, we estimate the amount of energy the HE can be transfer to the molecular vibrational modes using PBE as XC-functional in $3.07 \cdot 10^{-6}$ eV and $0.92 \cdot 10^{-6}$ eV for the desorption and the inner stretching mode respectively. Such values are very close to the one estimated with LDA (see Table S2) and therefore we can conclude that, apart from minor differences, the choice of the XC-functional seems not to affect the main findings of our study.
We also would like here to recall that we chose the adiabatic approximation to model the real time dynamics of the electrons. While it is known that this approximation give artifacts in model potentials upon interaction with light (which is not the situation simulated here), its use for propagating an initially-prepared non-stationary state in

realistic systems has given physically reasonable results and many important insights already.[13]

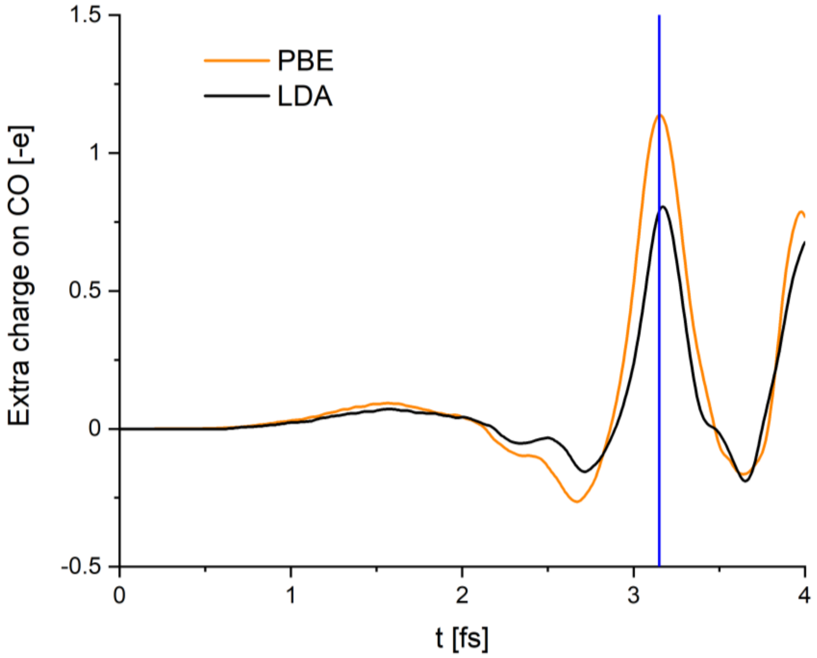

Figure S8. Amount of extra charges located on CO in the case of Ag-CO chain, as function of time in the case of simulations performed using PBE and LDA XC-functionals. The HE energy is 3.64 eV and 3.81 eV in the PBE and LDA cases respectively. Blue line indicates the actual HE injection on the absorbed molecule for the PBE-based simulation (i.e., 3.15 fs).

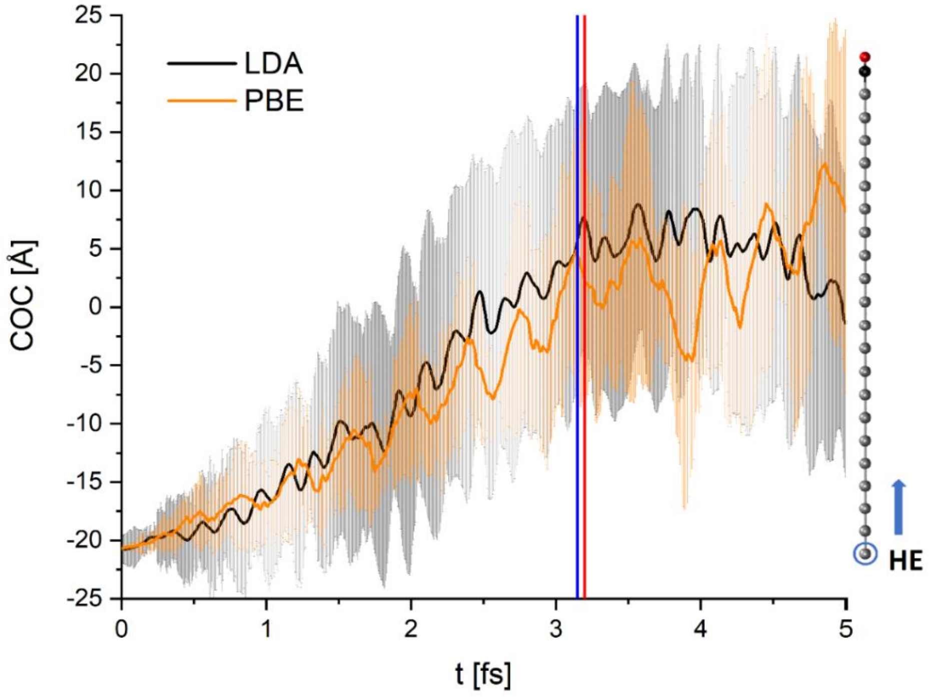

Figure S9. Time evolution of the COC for the Ag-CO system obtained with the PBE and LDA XC-functionals. The chain model on the right serves as a reference. Gray and orange vertical lines represent the uncertainty connected to the HE position. Blue and red lines indicate the actual HE injection on the absorbed molecule for the PBE and LDA-based simulation respectively.

Analysis on the forces developing on charged water molecule

To validate the results and observations made in the case of hot electron injection into water molecules, we studied how does an additional negative charge stimulates the development of forces acting on atomic nuclei.

To do this, we performed two separate ground-state DFT calculations, using as geometry the optimized geometrical structure of an isolated water molecule. One self consistent field calculation was made on the neutral system, the other included an extra -1 charge. We studied what forces develop on the various atoms and projected them onto the normalized vibrational displacement vectors for the bending and symmetric stretching motions (see eq. S4.2 and S4.3). These projected forces are collected in Table S3.

As can be seen from the values, the presence of a negative charge destabilizes the geometry of the molecule by generating new forces that cause atoms to move in accordance with the bending and symmetric O-H stretching modes. The latter in particular is the main activated vibrational mode, as in the Ag-H_2O case discussed in the main text.

The view discussed in the main text is therefore consistent with what can be estimated from charged systems of simple molecules.

Table S3. Projection of the forces over the bending and symmetrical stretching motions for neutral and -1 charged water molecule.

System net charge	$F_B^i \cdot 10^{-3}$ [a.u.]	$F_S^i \cdot 10^{-3}$ [a.u.]
0	-0.07	-0.1
-1	87	1200

Structural and geometrical parameters

In Table S4 we collected the structural parameters of the systems adopted in our simulations. All intramolecular bonds length are compatible with the theoretical values calculated for the species adsorbed on Ag (111)[7] and Au (111)[8] and are very similar to the ones of the free molecules in the gas phase.[14] The latter consideration remains valid for H_2O inner angle which measure 108.5° and 105.2° in the case of Ag and Au supported systems respectively.

Regarding the metal-metal distances within the chains, we found an average bond length of about 2.1±0.1 Å and 1.8±0.1 Å for Ag-Ag and Au-Au respectively. Such distances are considerably shorter than what is observed in the bulk (c.a. 2.9 Å) and in small metal aggregates.[15,16] We suggest that such an important rate of contraction is related to the monodimensional nature of the systems since, being the atoms highly uncoordinated, they tend to get closer and maximize their electronic clouds overlap. This represents one of the main limitations of our methodology that, rather than representing a realistic system, wants to qualitatively explore the effects occurring during the HE-molecule interaction through a simple, versatile and feasible strategy. Finally, we underline that we could not recover any stable relaxed geometry for the Au-N_2 system. It is indeed well known that N_2 does not attach on gold surfaces and can only be physiosorbed.[6] Therefore we impose as reference values for the Au-N and the N-N distance the one obtained in the case of silver chains, in order to have a direct comparison on the HE performances in the two cases.

Table S4. Bond lenght for the various M-X and X-Y bonds. M=Ag, Au; X=C, N, O and Y=O, N, H. d_{M-X} is the bond length values in our systems, while d^{L}_{M-X} are the theoretical values reported in the literature for the molecules adsorbed on the M (111) slab.[7,8] All values are given in Å.

M	X	d_{M-X}	d^{L}_{M-X}	X-Y	d_{X-Y}	d^{L}_{M-X}
Ag	C	1.96	2.12	C-O	1.12	1.16
	N	1.74	-	N-N	1.08	1.09
	O	1.62	1.47	O-H	0.97	0.98
Au	C	1.53	1.36	C-O	1.10	1.19
	N	1.74	-	N-N	1.08	1.09
	O	1.72	1.76	O-H	0.97	0.99

Description of C-EOM in the case of water molecule

The Classical Equation Of Motions (C-EOM) system for the water molecule is:

$$\begin{cases} m_O\ddot{z}_O(t) + k_{M-O}z_O(t) + 2k_{O-H}[z_O(t) - z_H(t)] = F_O^z(t) \\ m_H\ddot{y}_{H_1}(t) + k_{O-H}\, y_{H_1}(t) = F_{H_1}^y(t) \\ m_H\ddot{z}_{H_1}(t) - k_{O-H}\big[z_O(t) - z_{H_1}(t)\big] = F_{H_1}^z(t) \\ m_H\ddot{y}_{H_2}(t) + k_{O-H}\, y_{H_2}(t) = F_{H_2}^y(t) \\ m_H\ddot{z}_{H_2}(t) - k_{O-H}\big[z_O(t) - z_{H_2}(t)\big] = F_{H_2}^z(t) \end{cases}$$

Where m_O, m_H, are the masses of oxygen and hydrogen, k_{M-O}, k_{O-H} are the spring constants of the M-O (M=Au, Ag) bond and intramolecular O-H bond, $z_O(t)$, $\ddot{z}_O(t)$, are the positions and accelerations of oxygen given with respect to their initial values, $z_{H_1}(t)$, $z_{H_2}(t)$, $\ddot{z}_{H_1}(t)$, $\ddot{z}_{H_2}(t)$, $y_{H_1}(t)$, $y_{H_2}(t)$, $\ddot{y}_{H_1}(t)$, $\ddot{y}_{H_2}(t)$ are the projection of the two H atoms displacements and accelerations along the z and y axis respectively with respect to their initial values, $F_O(t)$ is the force acting on oxygen and $F_{H_1}^z(t)$, $F_{H_2}^z(t)$, $F_{H_1}^y(t)$, $F_{H_2}^y(t)$ are the projections of the forces acting on the two H atoms along z and y axis respectively at time t. As pictured in Figure S1, since the M-O bond is oriented along the z axis and the molecule extends on the z-y plane, the symmetry of the system imposes that the forces coming from the arrival of HE that act on the two hydrogens, have the same components on the z axis while have opposite components on the y axis which means that $F_{H_1}^y(t) = -F_{H_2}^y(t)$.

Bibliography

(1) Andrade, X.; Strubbe, D.; De Giovannini, U.; Larsen, A. H.; Oliveira, M. J. T.; Alberdi-Rodriguez, J.; Varas, A.; Theophilou, I.; Helbig, N.; Verstraete, M. J.; et al. Real-Space Grids and the Octopus Code as Tools for the Development of New Simulation Approaches for Electronic Systems. *Phys. Chem. Chem. Phys.* **2015**, *17*, 31371–31396.

(2) Tancogne-Dejean, N.; Oliveira, M. J. T.; Andrade, X.; Appel, H.; Borca, C. H.; Le Breton, G.; Buchholz, F.; Castro, A.; Corni, S.; Correa, A. A.; et al. Octopus, a Computational Framework for Exploring Light-Driven Phenomena and Quantum Dynamics in Extended and Finite Systems. *J. Chem. Phys.* **2020**, *152*, 1–32.

(3) Hartwigsen, C.; Goedecker, S.; Hutter, J. Relativistic Separable Dual-Space Gaussian Pseudopotentials from H to Rn. *Phys. Rev. B* **1998**, *58*, 3641–3662.

(4) IUPAC. International Union of Pure and Applied Chemistry https://iupac.org/what-we-do/periodic-table-of-elements/. Accessed in November 2021.

(5) Lykke, K. R.; Kay, B. D. Rotational Rainbows in the Inelastic Scattering of N_2 from Au(111). *J. Phys. Condens. Matter* **1991**, *3*, S65–S70.

(6) Meng, G.; Yin, R.; Zhou, X.; Jiang, B. Theoretical Study of Weakly Bound Adsorbates on Au (111): Tests on van Der Waals Density Functionals. *J. Phys. Chem. C* **2021**, *125*, 24958–24966.

(7) Chen, B. W. J.; Kirvassilis, D.; Bai, Y.; Mavrikakis, M. Atomic and Molecular Adsorption on Ag(111). *J. Phys. Chem. C* **2019**, *123*, 7551–7566.

(8) Santiago-Rodríguez, Y.; Herron, J. A.; Curet-Arana, M. C.; Mavrikakis, M. Atomic and Molecular Adsorption on Au(111). *Surf. Sci.* **2014**, *627*, 57–69.

(9) Frisch, M. J.; Trucks, G. W.; Schlegel, H. B.; Scuseria, G. E.; Robb, M. A.; Cheeseman, J. R.; Scalmani, G.; Barone, V.; Petersson, G. A.; Nakatsuji, H.; et al. Gaussian 09. 2010, p Revision B.01; Gaussian, Inc; Wallingford, CT, 201.

(10) Humphrey, W.; Dalke, A.; Schulten, K. VMD: Visual Molecular Dynamics. *J. Mol. Graph.* **1996**, *14*, 33–38.

(11) Ashcroft, N. W.; Mermin, N. D. *Solid State Physics*; Saunders College Publishing, Philadelphia, 1976.

(12) Perdew, J. P.; Burke, K.; Ernzerhof, M. Generalized Gradient Approximation Made Simple. *Phys. Rev. Lett.* **1996**, *77*, 3865–3868.

(13) Li, X.; Govind, N.; Isborn, C.; Deprince, A. E.; Lopata, K. Real-Time Time-Dependent Electronic Structure Theory. *Chem. Rev.* **2020**, *120*, 9951–9993.

(14) CRC Handbook of Chemistry and Physics. *Structure of Free Molecules in the Gas Phase*, 102nd Edit.; CRC Press, 2021.

(15) Fournier, R. Theoretical Study of the Structure of Silver Clusters. *J. Chem. Phys.* **2001**, *115*, 2165–2177.

(16) Miller, J. T.; Kropf, A. J.; Zha, Y.; Regalbuto, J. R.; Delannoy, L.; Louis, C.; Bus, E.; van Bokhoven, J. A. The Effect of Gold Particle Size on Au-Au Bond Length and Reactivity toward Oxygen in Supported Catalysts. *J. Catal.* **2006**, *240*, 222–234.
